# COVID-19 cynomolgus macaque model reflecting human COVID-19 pathological conditions

**DOI:** 10.1073/pnas.2104847118

**Published:** 2021-10-08

**Authors:** Emiko Urano, Tomotaka Okamura, Chikako Ono, Shiori Ueno, Satoshi Nagata, Haruhiko Kamada, Mahoko Higuchi, Mugi Furukawa, Wataru Kamitani, Yoshiharu Matsuura, Yoshihiro Kawaoka, Yasuhiro Yasutomi

**Affiliations:** ^a^Laboratory of Immunoregulation and Vaccine Research, Tsukuba Primate Research Center, National Institutes of Biomedical Innovation, Health and Nutrition, Tsukuba 305-0843, Japan;; ^b^Laboratory of Virus Control, Research Institute for Microbial Diseases, Osaka University, Osaka 565-0871, Japan;; ^c^Department of Infectious Diseases and Host Defense, Graduate School of Medicine, Gunma University, Maebashi 371-8511, Japan;; ^d^Laboratory of Antibody Design, National Institutes of Biomedical Innovation, Health and Nutrition, Osaka 567-0085, Japan;; ^e^Laboratory of Biopharmaceutical Research, National Institutes of Biomedical Innovation, Health and Nutrition, Osaka 567-0085, Japan;; ^f^Division of Virology, Department of Microbiology and Immunology, The Institute of Medical Science, The University of Tokyo, Tokyo 108-8639, Japan;; ^g^Department of Pathobiological Sciences, School of Veterinary Medicine, University of Wisconsin–Madison, Madison, WI 53706;; ^h^Department of Special Pathogens, International Research Center for Infectious Diseases, The Institute of Medical Science, The University of Tokyo, Tokyo 108-8639, Japan;; ^i^Division of Immunoregulation, Department of Molecular and Experimental Medicine, Mie University Graduate School of Medicine, Mie 514-8507, Japan

**Keywords:** COVID-19, SARS-CoV-2, nonhuman primate, elderly, underlying disease

## Abstract

The pandemic of severe acute respiratory syndrome coronavirus 2 (SARS-CoV-2) is a global threat to human health and life. A useful pathological animal model accurately reflecting human pathology is needed to overcome the COVID-19 crisis. In the present study, COVID-19 cynomolgus monkey models including monkeys with underlying diseases causing severe pathogenicity such as metabolic disease and elderly monkeys were examined. Cynomolgus macaques with various clinical conditions were intranasally and/or intratracheally inoculated with SARS-CoV-2. Infection with SARS-CoV-2 was found in mucosal swab samples, and a higher level and longer period of viral RNA was detected in elderly monkeys than in young monkeys. Pneumonia was confirmed in all of the monkeys by computed tomography images. When monkeys were readministrated SARS-CoV-2 at 56 d or later after initial infection all of the animals showed inflammatory responses without virus detection in swab samples. Surprisingly, in elderly monkeys reinfection showed transient severe pneumonia with increased levels of various serum cytokines and chemokines compared with those in primary infection. The results of this study indicated that the COVID-19 cynomolgus monkey model reflects the pathophysiology of humans and would be useful for elucidating the pathophysiology and developing therapeutic agents and vaccines.

## Significance

COVID-19 has shown severe pathogenicity in people with underlying diseases and in elderly people. It is necessary to elucidate the pathological conditions in patients with underlying diseases and in elderly patients. The use of appropriate animal models of COVID-19 is required for the development of pharmaceutical products; however, usually healthy young animals are used as experimental animals. Cynomolgus macaques with various clinical conditions and ages were infected with severe acute respiratory syndrome coronavirus 2 and there was a difference in pathological condition between young individuals and old-aged individuals with underlying diseases; therefore, the COVID-19 cynomolgus monkey model reflecting the pathophysiology of humans would be useful for elucidating the pathophysiology and developing therapeutic and prophylactic agents.

At the end of 2019, cases of infection with a novel coronavirus, later named severe acute respiratory syndrome coronavirus 2 (SARS-CoV-2), that causes various respiratory symptoms expanded globally from China. On 11 March 2020, the World Health Organization declared a pandemic status based on the spread of infection worldwide and increase in the number of deaths. The outbreak of SARS-CoV-2 has resulted in a miserable reality for global health and life, and infection cases are continuing to increase worldwide. A detailed understanding of pathological conditions and development of effective therapeutic agents are needed to overcome the pandemic of diseases caused by SARS-CoV-2 infection, which has been named COVID-19. A new strategy including the development of pharmaceutical products and the use of appropriate animal models reflecting the human pathogenesis of COVID-19 is required.

The common clinical features of COVID-19 are respiratory symptoms associated with pneumonia including fever, cough, myalgia, fatigue, dyspnea, and lymphopenia (1). COVID-19 symptoms range from no symptoms to severe symptoms. COVID-19 has shown severe pathogenicity in people with underlying diseases and in elderly people (2, 3). The period from onset of COVID-19 symptoms to death varies depending on the age of the patient and underlying disease status of the patient (4). Therefore, in addition to elucidating the pathological features in healthy individuals, it is necessary to elucidate the pathological conditions in patients with underlying diseases and in elderly patients.

Cynomolgus monkeys (CMs), which are common laboratory animals among nonhuman primates (NHPs), show various human-like characteristics, including higher brain functions, long life span, single pregnancy, and regular menstrual periods, which are not found in other experimental animals. In the present study, COVID-19 model CMs, including healthy young CMs, elderly CMs (23 to 30 y of age, equivalent to 69 to 90 y of age in humans), and CMs with underlying diseases including diabetes and hyperlipidemia were experimentally infected with SARS-CoV-2 as animal models reflecting human pathology.

## Results

### Changes in Clinical Symptoms in CMs after SARS-CoV-2 Infection.

**Fig. 1.**
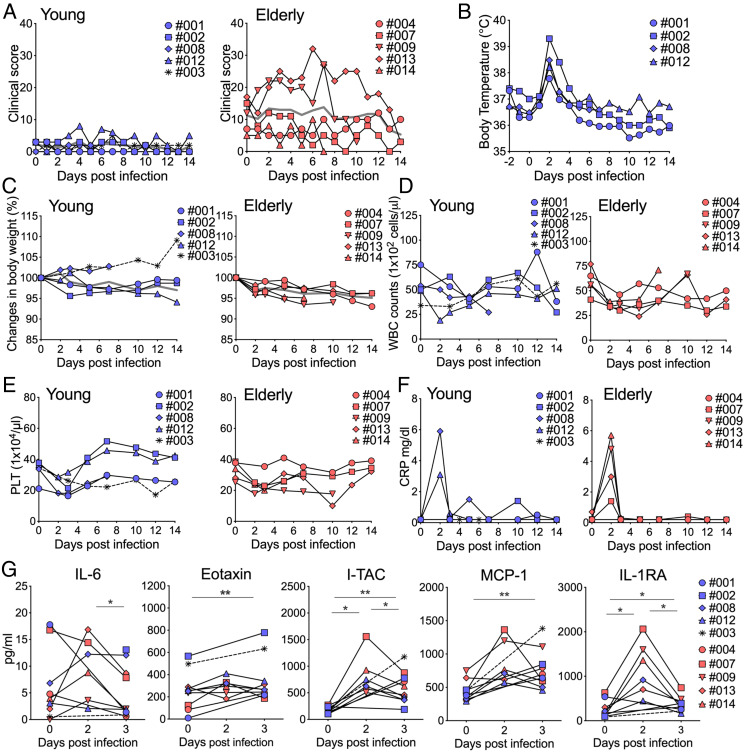
Clinical symptoms and biomedical changes in SARS-CoV-2–infected CMs. (*A*) SARS-CoV-2–infected animals were monitored for clinical signs and were individually scored daily in six categories: appearance, secretion, respiration, discharging, appetite, and activity (*Left*: young, *Right*: elderly). Body temperature was recorded by a data logger in young SARS-CoV-2–infected monkeys (*B*). Changes in body weight (*C*), white blood cell counts (*D*), platelets (*E*), and CRP (*F*) were examined at the indicated time points. The average values in the groups with the same inoculate doses are indicated by gray lines (*A* and *C*). Levels of 29 cytokines and chemokines in serum after infection were analyzed. Displayed cytokines and chemokines were increased in association with the infection (*G*) (blue: young, red: elderly). Statistical analyses between the indicated time points were performed using Wilcoxon test. *P < 0.05; **P < 0.01.

Two groups of CMs (a young group that included five CMs (three males and two females aged 3 to 9 y and an elderly group of five female CMs aged 23 to 29 y) were used in this study (Table 1). All of the young CMs were healthy with no apparent clinical symptoms (Table 1). Some CMs in the elderly group had underlying disease. CM #007 had diabetes mellitus and hyperlipidemia (HL). CM #009 was obese and had HL, and CM #013 also had HL and was in a prediabetic stage (Table 1). Nine CMs were infected with 1 × 10^6^ TCID_50_ (tissue culture 50% infectious dose) SARS-CoV-2 via a combination of intratracheal (IT) and intranasal (IN) routes, and one young CM, #003, was infected with 1/10 amount of virus (1 × 10^5^ TCID_50_) by IT administration (Table 2). Obvious clinical symptoms were not observed in young CMs, but scores for clinical signs in addition to age-related factors increased in the elderly group after SARS-CoV-2 infection (Fig. 1*A*). The main causes of the increases in scores were decreased appetite and decreased bowel movement. Considering the physical burden for elderly CMs, body temperature was monitored daily by an intraperitoneally embedded data logger in only four young CMs, and all of those four CMs had a fever at day 1 postinfection (p.i.). The elevated temperature gradually returned to normal within 1 to 3 d (Fig. 1*B*). No significant body weight loss was observed in any of the CMs; however, CMs in the elderly group showed a tendency for body weight loss during the initial stage of infection within 14 d (Fig. 1*C*). A decrease in white blood cells was observed in all SARS-CoV-2–infected CMs at 3 to 5 d p.i. (Fig. 1*D*). A decrease in platelets after infection has been reported in humans (5) and was also observed in the CMs (Fig. 1*E*). The inflammatory biomedical marker c-reactive protein (CRP) was elevated on day 2 p.i. but decreased rapidly on day 3 p.i. in both groups of CMs (Fig. 1*F*). The values showing functions of liver and related organs in serum were increased in some CMs, but there was no fixed tendency (*SI Appendix*, Fig. S1). Three young CMs and three elderly CMs were administered the same volume of phosphate-buffered saline (PBS) instead of the virus as a sham control group (Table 3). Clinical changes seen in SARS-CoV-2–infected animals were not observed in the PBS-administered sham group (*SI Appendix*, Fig. S2). The fractions of peripheral blood mononuclear cells (PBMCs) in both groups of monkeys were examined by flow cytometry analysis (*SI Appendix*, Fig. S3). The percentages of CD4^+^ T cells after infection were slightly increased in the two groups of animals. The percentage of CD8^+^ T cells decreased transiently after infection, but there was no significant difference between the indicated time points. CD20^+^ cells, which are B cells, decreased after infection and gradually returned to the preinfection level. CD159a^+^ (NKG2A^+^) natural killer (NK) cells showed great changes after infection. The percentages of NK cells decreased immediately after infection, and there was no significant difference between the elderly and young groups. Next, several cytokines and chemokines that have been suggested to be related to viral infection and pathophysiology were examined. Changes in one cytokine (interleukin [IL]-6), one cytokine-related protein (IL-1 receptor antagonist [IL-1RA]), and three chemokines (eotaxin, monocyte chemoattractant protein [MCP]-1, and I-TAC [CXCL11]) were observed immediately after infection in most CMs. Most of these cytokines and chemokines were transiently but significantly elevated in each animal compared to the levels between the indicated time points (Fig. 1*G*).

**Table 1.** Monkeys and characteristics

Age group and no.	Sex	Age, y	Origin	Weight, g	Glucose, mg/dL	HbA1c, %	Triglyceride, mg/dL	LDL/HDL ratio	Underlying disease
Young									
#001	M	3	Mix*	2,355	34		20		
#002	M	3	Mix†	2,295	41		59		
#008	F	7	Malaysia	2,915	36		24		
#012	F	8	Philippines	3,370	41		45		
#003	M	6	Philippines	3,645	42		19		
Elderly									
#004	F	23	Malaysia	3,385	51		31		
#007	F	30	Malaysia	3,345	88‡	6.5	316	2.50	DM, HL
#009	F	24	Indonesia	6,035	64	3.9	212	2.00	Obesity, HL
#013	F	29	Indonesia	4,845	98	4.6	203	1.57	HL, prediabetes
#014	F	27	Philippines	4,845	67		75		

M, male; F, female; DM, diabetes mellitus; LDL, low-density lipoprotein; HDL, high-density lipoprotein.

* Mixed with Malaysia and Indonesia.

† Mixed with Malaysia, Indonesia, and Philippine.

‡ #007: Glucose value was 203 mg/dL at the regular health examination a year before the experiment.

**Table 2.** Experimental design

Age group and no.	Infection dose, TCID_50_	Infection route	Days at Reinfection	Reinfection dose, TCID_50_	Reinfection route	Days at Necropsy
Young						
#001	1 × 10^6^	IT, IN	56	1 × 10^5^	IT	70
#002	1 × 10^6^	IT, IN	—			14
#008	1 × 10^6^	IT, IN	—			7
#012	1 × 10^6^	IT, IN	112	1 × 10^6^	IT	140
#003	1 × 10^5^	IT	—			14
Elderly						
#004	1 × 10^6^	IT, IN	56	1 × 10^5^	IT	70
#007	1 × 10^6^	IT, IN	56	1 × 10^6^	IT	63
#009	1 × 10^6^	IT, IN	—			10
#013	1 × 10^6^	IT, IN	—			14
#014	1 × 10^6^	IT, IN	—			7

**Table 3.** Sham group monkeys and characteristics

Age group and no.	Sex	Age, y	Origin	Weight, g	Glucose, mg/dL	Triglyceride, mg/dL	Underlying disease	Administration route
Young								
sy001	F	7	Indonesia	2,580	55	67		IT, IN
sy002	M	8	Malaysia	4,045	53	54		IT, IN
sy003	M	7	Malaysia	3,804	52	15		IT, IN
Elderly								
se001	F	23	Indonesia	2,720	47	80	Cataract	IT, IN
se002	F	23	Mix*	3,085	73	46		IT, IN
se003	F	26	Philippines	3,885	42	44		IT, IN

* Mixed with Indonesia and Malaysia.

### Detection of SARS-CoV-2 from Mucosal Swabs after Infection.

**Fig. 2.**
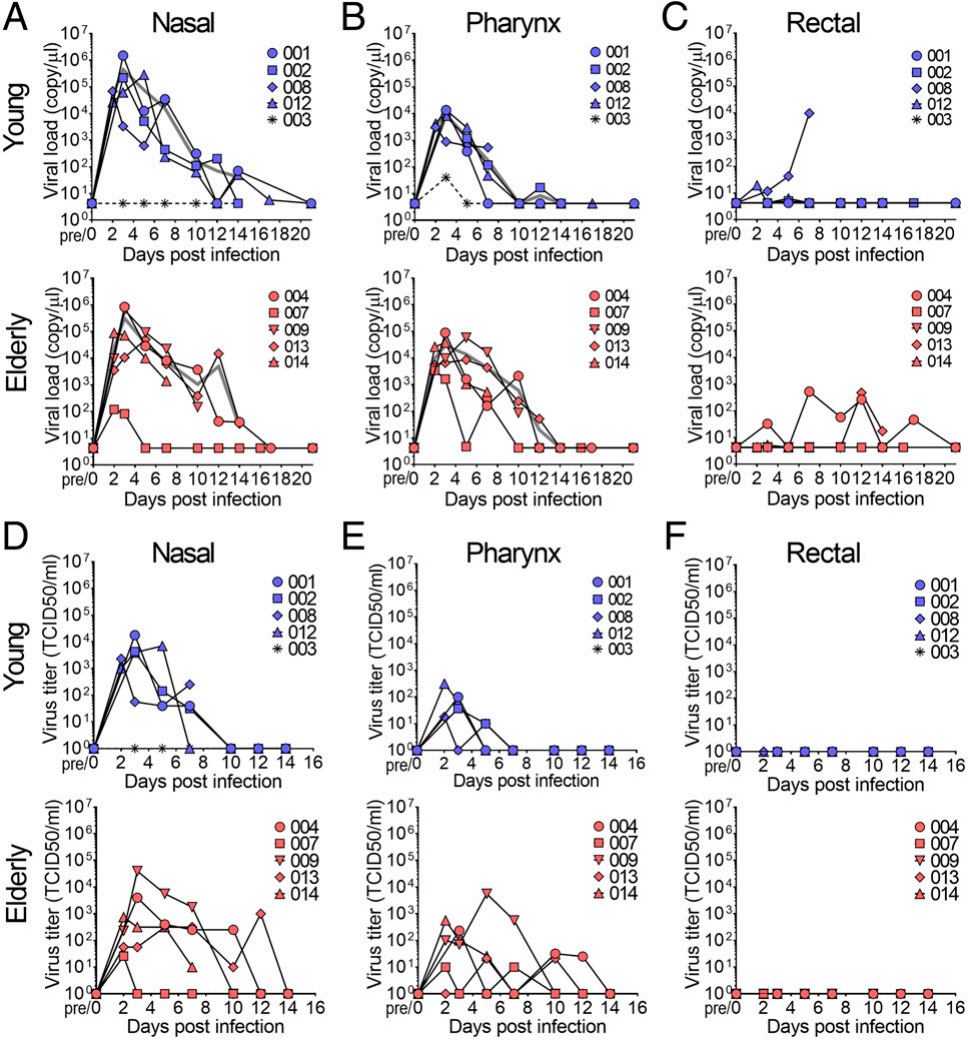
Determination of viral shedding by measurements of viral loads and viral titers in nasal, pharynx, and rectal swab samples. After inoculation of SARS-CoV-2, nasal (*A* and *D*), pharynx (*B* and *E*), and rectal (*C* and *F*) swab samples were collected at the indicated time points. Viral loads were determined by RT-PCR using RNA extracted from swab samples (*A–C*; blue: young, red: elderly). The average viral loads in the groups with the same inoculate doses are indicated by gray lines. Infectious viral titers in swab samples were determined by TCID_50_ using VeroE6/TMPRSS2 cells (*D–F*; blue: young, red: elderly).

RT-qPCR was carried out to detect viral RNA in nasal, pharynx, and rectal swab samples. The average viral RNA loads in 1 × 10^6^ TCID_50_-infected CMs are shown by gray lines in Fig. 2. Viral RNA in nasal swabs peaked at day 3 p.i. and the viral RNA peak in nasal swabs was higher than the peaks in pharynx and rectal swabs (Fig. 2*A*). In pharynx swabs, a substantially higher level of viral RNA was detected in the elderly group than in the young group, and the level decreased gradually until day 14 p.i. (Fig. 2*B*). In the young group, viral RNA peaked at the same time as that in the elderly group, but the amount of viral RNA detected in the young group decreased rapidly (Fig. 2*B*). When the viral titer was examined using the same swab samples, the difference between the young group and the elderly group became clearer (Fig. 2*D*). Infectious viruses were detected in nasal and pharynx samples for longer periods in the elderly monkeys than in the young monkeys (Fig. 2 *D* and *E*). Viral RNA was detected in rectal swabs from some CMs and in CM #004 for a longer period of time than in the other CMs; however, no infectious virus was detected (Fig. 2 *C* and *F*).

### Computed Tomography Analysis of the Lungs of SARS-CoV-2–Infected CMs.

**Fig. 3.**
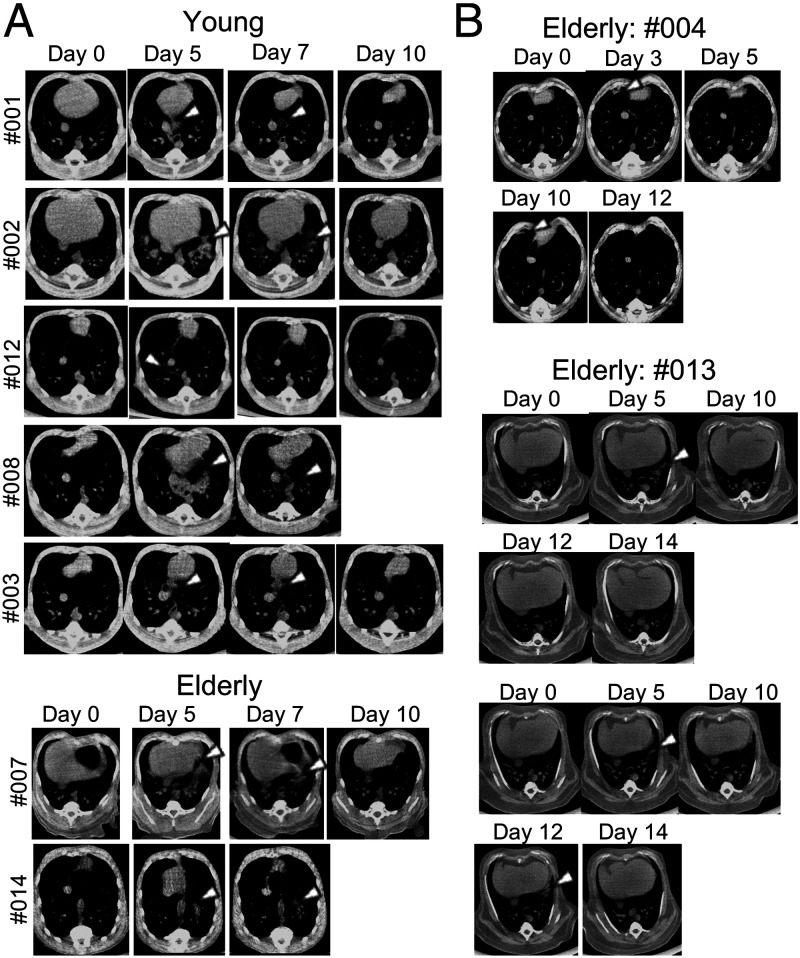
Pulmonary inflammation in SARS-CoV-2–infected CMs. Chest CT images were obtained during the experimental period. Pulmonary inflammations were observed and mostly peaked at 5 d p.i. in both the young and elderly groups (*A*). Images of inflammation were obtained for a longer period in elderly animals (*B*). The white arrow indicates a representative inflammation image of pneumonia.

An elderly CM (#009) with obesity was excluded from computed tomography (CT) analysis due to the lack of a clear chest image, probably caused by its obesity. Pneumonia was observed by CT in all CMs. Typical inflammatory images were obtained in all CMs and two elderly CMs (#007 and #014) from 3 d p.i., and the inflammation peaked at 5 d after infection (Fig. 3*A*). In these CMs, the pneumonia disappeared within 10 d after infection. CM #003 that was infected with a low dose of virus also developed pneumonia even though viral RNA was detected only in the pharynx at day 3 p.i. without detection of infectious virus (Fig. 2). Although severe inflammatory images were not obtained in the elderly CMs, pneumonia tended to be observed in the elderly CMs for a longer period and it developed in different locations at different time points. In the elderly animals (#004 and #013), recurrence of pneumonia was confirmed occasionally by day 12 (Fig. 3*B*).

### Virus Quantification in Systemic Organs and Virus Expression in Lung.

Two young CMs (#002 and #008) and three elderly CMs (#009, #013, and #014) were subjected to pathological dissection at 7 to 14 d after infection, and the results of RT-qPCR for those CMs are shown in *SI Appendix*, Fig. S4. Loads of the virus detected by RT-qPCR in organs were measured by a previously reported method for assessing vaccine efficacy so that all individuals could be compared equally (6, 7). Samples were obtained from various organs for assessment of SARS-CoV-2 localization and were scaled for the weight of each piece of organ. To collect fragments of the same sizes from the same sites of the organs of each animal, a template using a biopsy punch instrument was placed over the organ. At 7 d after infection, the virus was detected in various tissues and organs by qPCR of the N gene in both a young CM (#008) and an elderly CM (#014). Subgenome RNA (sgRNA) is thought to reflect the viral replication in infected cells (8, 9). When subgenome qPCR was performed, higher positive reactions were detected in the lungs in the elderly CM (#014) than in the young CM (#008) and sgRNA was also detected in mediastinal lymph nodes of CM #014 (*SI Appendix*, Fig. S4*A*). Similarly, the N gene was positive in various tissues of the elderly CM #009 at day 10; however, sgRNA was positive only in one part of the lung that showed the largest N gene copy number (*SI Appendix*, Fig. S4*B*). At 14 d after infection, both CMs (young #002 and elderly #013) showed positive reactions in qPCR of the N gene. Positive reactions were mainly observed in the lungs at the inoculation site in the young CM (#002), whereas positive reactions were observed in many organs including digestive organs in the elderly CM (#013) (*SI Appendix*, Fig. S4*B*). Results of subgenome PCR were negative in all organs at 14 d p.i. In histopathological examinations, pneumonia was confirmed in lung tissues of all infected monkeys, and the virus was also found in epithelial cells by immunohistological examinations (*SI Appendix*, Fig. S4*C*).

### Reinjection of SARS-CoV-2 in CMs.

**Fig. 4.**
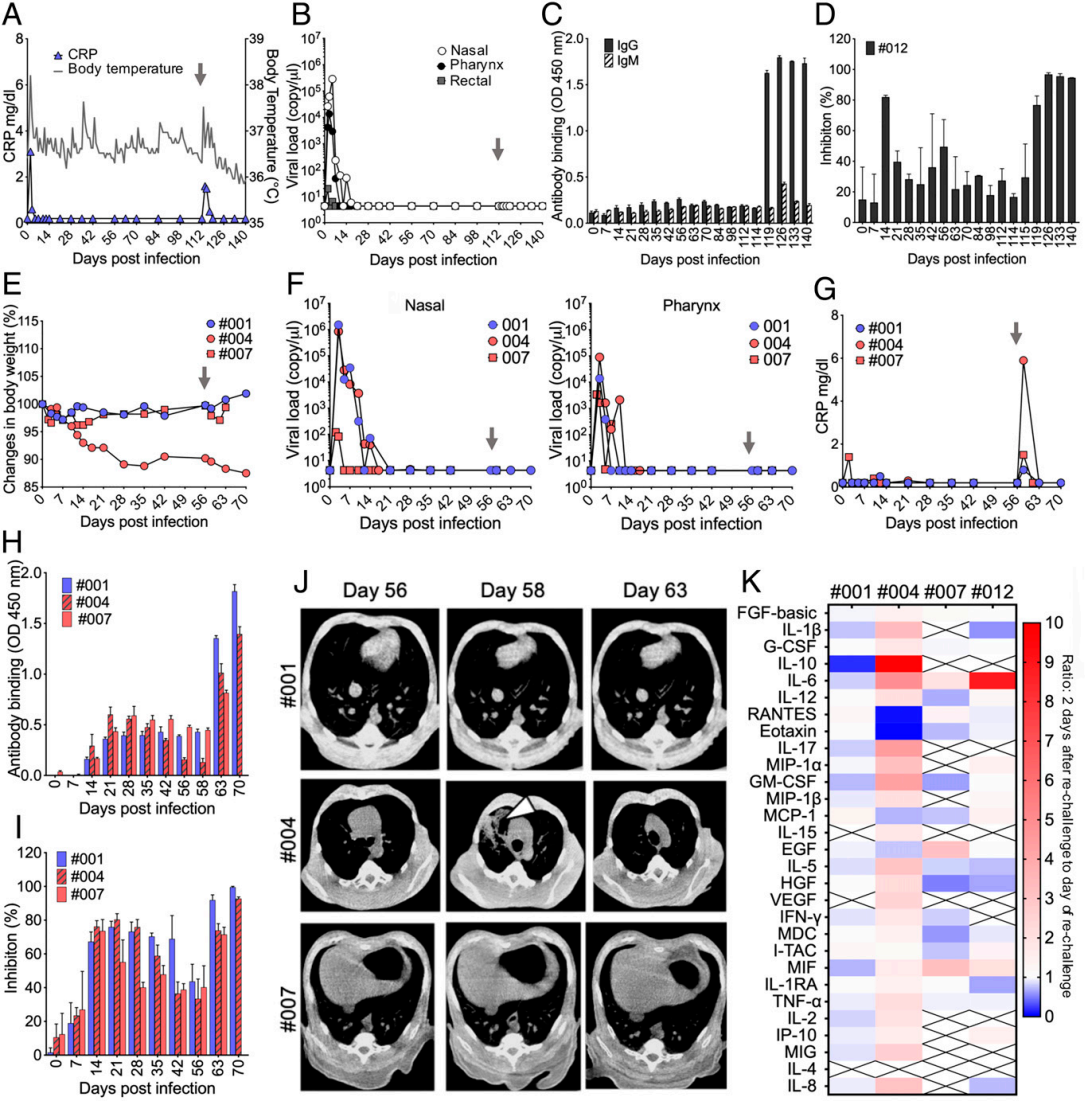
Prolonged analysis of clinical symptoms, viral shedding, Ab responses, and immunological responses in SARS-CoV-2–rechallenged animals. CM #012 was reinoculated with SARS-CoV-2 at 112 d after the initial infection. Changes in body temperature (*A*: gray line) and CRP (*A*: blue triangle) were monitored throughout the experiment period. Viral loads from nasal, pharynx, and rectal swabs were determined by RT-PCR (*B*). The gray allow indicates the time of reinoculation. Antigen-specific Ab responses were analyzed using CM #012 plasma samples. RBD-specific IgG and IgM Abs were measured by ELISA using 1:100-diluted plasma (*C*). Representative data are shown as means and SDs from an experiment done in triplicate. nAb titer in 1:100-diluted plasma was determined by using SARS-CoV-2 S protein pseudotyped VSV (*D*). Data are shown as means and SDs from two independent experiments done in triplicate. Three animals were reexposed to SARS-CoV-2 at 56 d after the initial infection. Changes in body weight (*E*), swab sample viral loads (*F*), and CRP (*G*) were examined at the indicated time points. The gray arrow indicates the time of reinoculation. RBD-specific IgG responses (*H*) and nAb titers (*I*) in 1:100-diluted plasma were determined throughout the experimental period. Representative data are shown as means and SDs from an experiment done in triplicate. Chest CT images were obtained on the day of rechallenge (day 56), 2 d after rechallenge (day 58), and 7 d after rechallenge (day 63) (*J*). The white arrow indicates a representative inflammation image of pneumonia. Changes in the levels of cytokines and chemokines in serum at the time of the second infection (*K*). The heat map shows ratio of the levels of cytokines and chemokines at 2 d after rechallenge (day 58 or day 114) to the levels of those at the day of rechallenge (day 56 or day 112).

Some CMs were then subjected to reinfection with SARS-CoV-2 (Table 2). To determine whether the remaining antibodies (Abs) that were induced after the initial infection directly contribute to the protection against reinfection, one young CM (#012) was intratracheally rechallenged with the same dose of SARS-CoV-2 as that used for the initial infection (1 × 10^6^ TCID_50_) at 112 d p.i., when the level of the receptor binding domain (RBD) of SARS-CoV-2–specific Ab titer and neutralizing Ab (nAb) activity had decreased to the levels before infection. The level of CRP and body temperature transiently increased after reinfection (Fig. 4*A*), and viral RNA from swab samples was below the detection limit (Fig. 4*B*); however, when a loop-mediated isothermal amplification (LAMP) kit was used, Tt values from pharynx samples using a double amount of RNA as a template were 22:24 on day 114 and 31:38 on day 115. RBD-specific Ab and nAb levels immediately increased after reinfection (Fig. 4 *C* and *D*). Pneumonia was not observed by CT analysis after injection of the virus in this CM. Some virus-related clinical symptoms were observed, but active viral replication and related disease were not confirmed. In addition, a differential infection experiment with a short period after the initial infection (56 d p.i.) was conducted using three CMs having low levels of RBD-specific Ab and nAb. The young CM (#001) and one of the elderly CMs (#004) were inoculated with a 1/10 amount of the virus used in the initial infection (1 × 10^5^ TCID_50_) since low-dose virus-infected CM #003 developed pneumonia, and the other elderly CM (#007) was injected with the same dose of the virus as that used in the initial infection (1 × 10^6^ TCID_50_). CM #004 gradually lost weight after the initial infection and lost further weight after reinoculation, and temporary weight loss just after the second infection was observed in other CMs (Fig. 4*E*). SARS-CoV-2 was not detected by RT-PCR in swab samples after readministration in any of the CMs (Fig. 4*F*), and the level of CRP showed transient increase after reinoculation (Fig. 4*G*). RBD-specific Ab and nAb levels immediately increased after reinfection in all CMs (Fig. 4 *H* and *I*). Surprisingly, the elderly CM with continuous weight loss (#004) showed temporary pneumonia by CT analysis at 2 d after reinoculation (Fig. 4*J*). The fractions of PBMCs in reinfected monkeys were also analyzed after rechallenge with the virus (*SI Appendix*, Fig. S5). The percentages of CD4^+^ T cells in three monkeys (#001, #007, and #012) for which CT imaging showed no pneumonia transiently increased, whereas CD8^+^ T cells decreased transiently after rechallenge with the virus (*SI Appendix*, Fig. S5 *A* and *B*). Interestingly, monkey #004 showing pneumonia and sustained weight loss showed the opposite changes after the second infection. The percentage of CD4^+^ T cells decreased and the percentage of CD8^+^ T cells increased after reinfection (*SI Appendix*, Fig. S5 *A* and *B*). A consistent tendency on changes for CD20^+^ cells and NK cells was not observed (*SI Appendix*, Fig. S5 *C* and *D*). The levels of five cytokines and chemokines were elevated at the time of initial infection (Fig. 1), but changes in more cytokines were found than those at the time of initial infection. In CM #004, which showed pneumonia, the levels of 24 of the 29 cytokines and chemokines were increased after reinoculation, and the other three CMs that did not show pneumonia showed increases in 4 (#001), 5 (#007), and 8 (#012) cytokines and chemokines (Fig. 4*K*). Viral sgRNA was not detected in several tissues and organs at necropsy. In the elderly CMs, some blood biochemical values increased after reinoculation of the virus (*SI Appendix*, Fig. S6). These results indicated that pneumonia that occurred after virus reinfection may not have been induced by direct tissue destruction by the virus and that it may have been caused by host immune responses.

The results of this study suggested that the COVID-19 CM model reflects the pathology of humans, and the results showing that the pathology is very complicated especially in elderly animals and animals with underlying diseases are considered to be very important.

## Discussion

The establishment of appropriate animal models is essential for overcoming the COVID-19 tragedy. The CM is considered to be one of the most suitable animals that reflect the pathological conditions of humans. In the present study, we established animal models of COVID-19 that may reflect human pathology by using healthy CMs as well as CMs of advanced age and with pathological conditions such as metabolic diseases.

The pathology of COVID-19 ranges from no symptoms to mild to severe symptoms to fatal symptoms (10, 11). Advanced age and various underlying diseases have been shown to be associated with an increased risk of death (12). The period from the occurrence of symptom to death was reported to be shorter in patients over 70 y of age than in patients less than 70 y of age (4). Several NHP models of COVID-19 have been reported (13–19), and there have also been some reports on an aged monkey model (14, 15, 17). Unlike the models in those studies, the models in our study included not only elderly monkeys of more than 23 y of age but also monkeys with metabolic diseases such as diabetes and HL, which have become problems as underlying diseases. In our study, the viral load in pharyngeal swabs was higher in the aged macaques than in the young macaques, and both viral RNA and infectious virus were detected in nasal and pharyngeal swabs for a long period of time in the aged macaques, as has been reported for humans (Fig. 2 *A–F*) (20).

The viral genome was detected in not only the respiratory region but also in lymph nodes, major organs, and digestive tissues at day 7 or later (*SI Appendix*, Fig. S4). Virus infection in intestinal enterocytes has been reported by several groups (21–23). In accordance with the fact that viral RNAs are often detected in feces of patients with COVID-19, viral RNAs were detected in some CMs. On the other hand, sgRNA was not detected in regions other than the respiratory region in CMs tested in this study. It is likely that the virus can infect intestinal tissue but that its replication efficacy is not equivalent to that in the respiratory region.

Elucidation of how adaptive immunity in patients is induced by SARS-CoV-2 natural infection is critical for establishing a vaccine strategy, serologic therapies, public health control, and prediction of infection. Rechallenging of NHP models has been reported, and it has been shown that protective immunity in NHPs infected with SARS-CoV-2 is induced against reexposure to SARS-CoV-2 (24, 25). PCR repositivity has been reported in human patients who recovered (26–28) and reinfection with SARS-CoV-2 has been confirmed in patients who recovered (reinfection tracker, https://bnonews.com/index.php/2020/08/covid-19-reinfection-tracker/) (29–32). Reinfection cases with symptoms have recently been reported in the United States and Ecuador (33, 34). The results of the present study rule out the possibility of reinfection; however, quite different and interesting results were obtained. Although some clinical symptoms such as increased body temperature and increased level of CRP were transiently observed after the second infection, all of the monkeys showed negative results of PCR in swab samples after rechallenging with SARS-CoV-2. Interestingly, in one older CM (#004) that was also negative by PCR, CT images of pneumonia showed aggravation after reinoculation with the virus (Fig. 4*J*). A serological test for CRP also showed inflammation (Fig. 4*G*). At the time of initial infection of the CMs, production of IL-6, eotaxin, IL-1RA, I-TAC, and MCP-1 was observed in the infected CMs, and transient increases in the levels were observed (Fig. 1*G*). However, levels of 24 of the 29 cytokines and chemokines that were measured were increased transiently in serum of a reinjected CM with pneumonia (#004; Fig. 4*K*). In addition, changes in the fraction of PBMCs in CM #004 showed a tendency opposite to that in other CMs that did not develop pneumonia after reinoculation of the virus (*SI Appendix*, Fig. S5). Production of Abs rapidly increased after the rechallenge in CM #004 (Fig. 4 *H* and *I*), indicating that the adaptive immune system, at least in the Ab production pathway, worked similarly to that in other CMs reinoculated with SARS-CoV-2 that did not have pneumonia and in which viral propagation was suppressed. Furthermore, the titer of RBD-binding Abs and neutralizing activities were consistently correlated in rechallenged CMs, suggesting that it was unlikely that Ab-dependent enhancement occurred in rechallenged CM #004 with pneumonia (35, 36). Although future immunological analysis is needed, these results indicate that the pathogenesis of COVID-19 is not due to direct damage caused by SARS-CoV-2. Biological disorders caused by the production of large amounts of cytokines and chemokines, the so-called cytokine storm (CS) or cytokine release syndrome (CRS), have also been concerned in COVID-19 (37–39).

In the present study, no severe or fatal symptoms caused by SARS-CoV-2 infection similar to those in humans were observed except for in one elderly CM (#004), and no clear answer could therefore be obtained regarding the relationship between CS or CRS and pathological condition; however, it is important to investigate the pathological conditions in this model for future research. In fact, it has been reported that IL-6 (2, 40, 41), eotaxin (42), IL-1RA (43), I-TAC (44), and MCP-1 (45), which were elevated in CMs after the initial infection, are also involved in aggravation in humans, and our CM COVID-19 model could be used in future pathological studies related to these factors. In addition, no significant differences in CT inflammatory images between young and elderly monkeys were obtained by CT analysis, but inflammation was observed in multiple locations in elderly monkeys, consistent with the results of study showing that the incidence of multiple ground-glass opacity was higher in elderly patients than in young patients (46–48). Moreover, the duration of inflammation in the lungs tented to be longer in the elderly CMs than in the young CMs, which is also consistent with a comparison between patients with severe symptoms and patients with nonsevere symptoms (49). Since there was no significant difference between those two groups, the accumulation of data using an NHP model will be very important for understanding the variations of pathological conditions caused by SARS-CoV-2.

Although vaccination has been started in many countries as a countermeasure against COVID-19, there are many issues regarding this disease that need to be elucidated, and a useful primate model is still necessary. In the present study, COVID-19 CM models that reflect the pathology of humans have shown the potential to contribute to detailed elucidation of the pathology and to the development of vaccines and therapeutic agents.

## Materials and Methods

### Viruses and Cells.

SARS-CoV-2 was isolated and propagated as previously described and used for the infection experiment (50). The virus used for rechallenge was original virus stock propagated in Vero/TMPRSS2 cells cultured with 2% fetal bovine serum (FBS) in Dulbecco’s modified Eagle’s medium (DMEM) at 37 °C for 3 d. Vero/TMPRSS2 cells were obtained from JCRB Cell Bank and were maintained in 5% FBS DMEM supplemented with 1 mg/mL G418 (Roche). VeroE6/TMPRSS2 (P13) cells were obtained from JCRB Cell Bank and were maintained in 7% FBS DMEM supplemented with 1 mg/mL G418. All cells were cultured at 37 °C in a humidified 5% CO_2_ atmosphere. The TCID_50_ of the virus was measured using VeroE6/TMPRSS2 cells.

### Animals.

CMs housed at the Tsukuba Primate Research Center (TPRC), National Institutes of Biomedical Innovation, Health and Nutrition (NIBIOHN, Ibaraki, Japan) were used in this study after approval by the Committee on the Ethics of Animal Experiments of NIBIOHN in accordance with the guidelines for animal experiments at NIBIOHN. The CMs used in this study are listed in Tables 1 and 3. All of the animals were negative for B virus, simian immunodeficiency virus, simian T cell leukemia virus, and *Mycobacterium*. The animals were handled under the supervision of the veterinarians in charge of the animal facility.

### SARS-CoV-2 Infection and Sampling Procedures.

The animals were housed in animal biosafety level 3 (ABSL3) facilities at the TPRC of NIBIOHN during the experimental period and were monitored throughout the study for physical health and clinical assessment. The clinical status of CMs was scored daily in six categories: appearance (skin and fur; 0 to 10), secretion (nose, mouth, eyes; 0 to 5), respiration (0 to 15), discharging (feces and urine; 0 to 10), appetite (food intake; 0 to 10), and activity (0 to 15) (51). A data logger was embedded intraperitoneally into young CMs to monitor temperature at the same time every day. CMs were inoculated with SARS-CoV-2 under anesthesia in a BSL3 room via a combination of IT (900 or 1,000 μL) and IN (50 μL per nostril) routes with a total of 1 × 10^6^ TCID_50_ virus. Sham control animals were administrated with same volume of PBS instead of the virus. CM #003 was inoculated by only intratracheally with 1 × 10^5^ TCID_50_ virus. All of the animal experiments were conducted under anesthesia conditions for taking blood and swab samples and for CT scanning at each of the collection points. Rechallenge was performed for CM #001 and CM #004 with 1 × 10^5^ TCID_50_ virus for CM #012 and CM #007 with 1 × 10^6^ TCID_50_ virus intratracheally.

### Micro-CT imaging.

Each animal was placed under anesthesia (7.5 mg/kg ketamine⋅HCl and 3 mg/kg xylazine). CT images were obtained (80 kV, 400 CT μA, field of view: 160 mm, scan time: respiratory-gated 8 min). All CT scans were performed in a BSL3 facility using a 3D micro-CT scanner system (Cosmo scan CT AX; Rigaku Corporation). After scanning, the lung images were reconstructed by using CosmoScan Database software of the micro-CT scanner (Rigaku Corporation). Slices of the third, sixth, and ninth thoracic vertebrae, including the upper, middle, and lower lung areas, respectively, were selected. The images were analyzed by using a Cosmo scan CT viewer (Rigaku Corporation).

### RNA Extraction and qRT-PCR.

Nasal, pharynx, and rectal swab samples were collected in 1 mL DMEM supplemented with 100 U/mL penicillin and 100 mg/mL streptomycin (Nacarai Tesque). RNA was extracted from swab samples using a QiaAmp Viral RNA kit (Qiagen) according to the manufacturer’s instructions. For the detection of viral RNA, 5 μL of extracted RNA was subjected to one-step real-time RT-PCR using a QunatiTect Probe RT-PCR (Qiagen) kit on a Light-Cycler 480 II (Roche Diagnostics). The following primer and probe sets were used: forward primer 5'-AAATTTTGGGGACCAGGAAC-3' and reverse primer 5'-TGGCAGCTGTGTAGGTCAAC-3' with probe FAM-5'-ATGTCGCGCATTGGCATGGA-3'-BHQ1. The reaction conditions of RT-PCR were 50 °C for 30 min (reverse transcription) and 95 °C for 15 min (activation of the polymerase), 45 cycles of 15 s at 95 °C (denaturation) followed by 60 s at 60 °C (annealing and extension). LAMP assay was tested using 10 μL of extracted RNA form swab samples by Loopamp SARS-CoV-2 detection kit (Eiken Chemical Co.) according to the manufacturer’s instructions.

### Determination of the Viral Distribution In Vivo.

The viral distribution was examined and quantified at necropsy. Samples were collected from the same place and the same position of each lung lobe, tissues, and organs using a biopsy-punch instrument (5 mm; Kaijirushi). All of the samples collected from tissues and organs were placed in RNAlater solution (Invitrogen) and then each weight-scaled piece of a sample was subjected to RNA extraction. Tissue samples were homogenized by MagNA Lyser Green Beads with MagNA Lyser, and RNA was extracted using a MagNA Pure Compact RNA Isolation Kit or a High Pure RNA Tissue kit (Roche). Real-time PCR was performed according to a previous report (52). The extracted RNAs were subjected to one-step real-time PCR using a THUNDERBIRD Probe One-step qRT-PCR Kit (Toyobo); the reaction was run on a StepOne Real-Time PCR System (Applied Biosystems). To quantify N messenger RNA, we used the primer pair N_Sarbeco_F1 (5'-CACATTGGCACCCGCAATC-3') and N_Sarbeco_R1 (5'-GAGGAACGAGAAGAGGCTTG-3') and the FAM-TAMRA–labeled specific probe N_Sarbeco_P1 (FAM-ACTTCCTCAAGGAACAACATTGCCA-TAMRA). Cycling conditions were 95 °C for 1 min followed by 40 cycles of 95 °C for 15 s and 58 °C for 45 s. The viral RNA levels (equivalent TCID_50_) were calculated by using extracted RNA from virus known TCID_50_ titer as a standard curve. sgRNA was detected using forward primer 5'-CGATCTCTTGTAGATCTGTTCTC-3' and reverse primer 5'-ATATTGCAGCAGTACGCACACA-3' with the probe FAM-5'-ACACTAGCCATCCTTACTGCGCTTCG-3'-BHQ1. The reaction conditions were 50 °C for 30 min, 95 °C for 15 min, and 45 cycles of 15 s at 95 °C followed by 60 s at 60 °C.

### Histopathology and Immunohistochemistry.

Specimens were immersed in 4% paraformaldehyde solution and embedded in paraffin. For immunostaining of specimens, deparaffinized thin sections (4 μm in thickness) were incubated with trypsin solution for 30 min at 37 °C. The sections were then treated with 0.3% H_2_O_2_ to quench endogenous peroxidase activity and were washed extensively in Tris-buffered saline. After a blocking step, the sections were incubated with mouse anti–SARS-CoV-2 N Ab (R&D, MAB10474, 1:1,000). For detection of N protein, primary Abs were detected with biotinylated goat anti-mouse immunoglobulin G (IgG) and avidin–biotin peroxidase complex (Vector Laboratories) by using diaminobenzidine/H_2_O_2_.

### Detection of Anti–SARS-CoV-2 Abs.

Levels of SARS-CoV-2 RBD-specific Abs in plasma were measured by an enzyme-linked immunosorbent assay (ELISA) using purified RBD protein. A DNA sequence encoding the RBD1 region (amino acids 319 to 541) of the spike protein of SARS-CoV-2 (GenBank accession number QHD43416.1) was designed by codon optimization, synthesized, and subcloned into a pcDNA3.1-based expression vector with the Fc region of human IgG1 at the C terminus. For RBD-Fc expression, the plasmid was transfected into the Expi293F cells using Expi293 Expression System (Thermo Fisher Scientific) and the cell culture supernatants were harvested. The produced RBD-Fc fusion protein was purified by affinity chromatography using a Protein A HP column (Cytiva). A 96-well flat plate was coated with 100 μL of 0.1 μg/mL RBD-Fc overnight at 4 °C and blocked with 1% bovine serum albumin/PBS for 1 h at 37 °C. Plasma samples at 1:100 dilutions were placed in each well and incubated overnight at 4 °C and then incubated with a 1:10,000-diluted anti-monkey IgG horseradish peroxidase (Nordic Immunology) or a 1:10,000-diluted anti-monkey IgM (Nordic Immunology) for 1 h at room temperature. The reaction was developed by adding a TMB substrate (Dako) and halted by adding a stop solution. The absorbance at 450 nm was read using an iMark plate reader (Bio-Rad). Assays were performed in triplicate in each experiment.

### Pseudotyped SARS-CoV-2 Neutralization Assay.

 To generate pseudotype vesicular stomatitis viruses (VSVs) bearing the SARS-CoV-2 S protein, we transfected the expression plasmid pCAGG-pm3-SARS2-S_Hu_-d19 encoding SARS-CoV-2 S protein with a deletion of C-terminal 19 amino acids, because it has been reported that partial deletion of the C-terminal cytoplasmic domain allowed efficient incorporation into VSV particles (53). Codon-optimized (f or human cells) SARS-CoV-2 S complementary DNA (GeneArt Gene Synthesis, Thermo Fisher Scientific) based on S protein of the SARS-CoV-2 strain TY-WK-5212020 (isolated by NIID) with a deletion of C-terminal 19 amino acids (amino acids 1 to 1254) was amplified and cloned into the pCAGG-pm3 vector and was designated as pCAGG-pm3-SARS2-S_Hu_-d19. A SARS-CoV-2 spike-pseudotyped VSV was prepared as described previously (54). Plasma was heat-inactivated by incubation at 56 °C for 30 min with 1% CHAPS (final concentration). Then, the SARS-CoV-2 pseudotyped virus was preincubated with 100-fold-diluted sera for 1 h at 4 °C and inoculated with VeroE6/TMPRSS2 cells. The cells were harvested 24 h later and luciferase activity was measured.

### Analysis of Peripheral Blood Lymphocyte Populations.

Peripheral blood lymphocyte populations were analyzed using cryopreserved PBMCs. A fixable near-infrared dead-cells stain kit (Invitrogen) was used to exclude dead cells from the analysis. Then cells were stained using the following monoclonal Abs: anti-CD3 (clone SP34-2, BV650; BD), anti-CD4 (clone L200, PerCP; BD), anti-CD8 (clone DK25, APC; Dako), anti-HLA-DR (clone G46-6, BV786; BD), anti-CD45 (clone D058-1283, BV395; BD), anti-CD20 (clone 2H7, Alexa 700; BD), and anti-CD159a (clone Z199, PC7; Beckman Coulter). Stained cells were fixed with 1% freshly prepared paraformaldehyde for at least 2 h and then analyzed in a FACS LSRFortessa X-20 flow cytometer (BD). Data were analyzed using FlowJo (BD) software.

### Serum Biochemical Analysis and Cytokine and Chemokine Profiles.

Cryopreserved serum was used for blood biochemical analysis. The items (CRP, aspartate aminotransferase, alanine aminotransferase, lactate dehydrogenase, alkaline phosphatase, and blood urea nitrogen) were determined in a BSL3 laboratory by using the Fuji DRI-CHEM system (Fujifilm). The profiles of cytokines and chemokines in sera were analyzed using a cytokine monkey magnetic 29-plex panel for Luminex platform (Invitrogen, Thermo Fisher Scientific) according to the manufacturer’s protocol. Beads were washed with 2% paraformaldehyde solution before reading, and then signals were detected and analyzed using a Luminex bio-plex system (Luminex) with Bio-Plex Manager 5.0 software (Luminex).

### Statistical Analysis.

Statistical analyses were performed using GraphPad Prism 7 software and *P* < 0.05 was considered significant. Comparison between young and elderly groups was performed two-tailed Mann–Whitney *U* test. Changes of cytokines and chemokines was analyzed by two-tailed Wilcoxon test.

## Data Availability

All study data are included in the article and/or *SI Appendix*.
